# Health trends and health indicators

**DOI:** 10.1186/1753-6561-7-S4-S6

**Published:** 2013-08-23

**Authors:** Marti G Parker

**Affiliations:** 1 Aging Research Center, Karolinska Institute and Stockholm University, Stockholm, Sweden

## 

Among the myriad of indicators available for measuring health, which ones are most appropriate for following health trends over time in the elderly population? Health in older people is often characterised by the simultaneous presence of multiple conditions, with or without disease, which cannot be cured. Morbid conditions interact with the environment and with the ageing process in ways we do not yet understand. The biomedical model of health is not enough to describe and analyse health in old people. We must complement the model with models of health as multi-dimensional and contextual, that is, consider the impact of the social and physical environment.

When we attempt to measure changes in health over time, it can be difficult to discern what is change in health and what is contextual change. For example, living conditions, access to care, definitions and awareness of disease, and expectations change over time and affect prevalence rates of health indicators.

There are two main reasons to study trends: to monitor and estimate needs for care, and to understand the forces and factors driving population health. To estimate needs for, e.g., home help or institutionalisation, indicators that encompass context may be most useful. For example, walking ability is contingent on access to walking aides. As aides have become better and more accessible, the needs for help with shopping may decrease, regardless of ability to walk without aides. To estimate needs for, e.g., orthopaedic care, indicators of range of motion, ability to kneel, and pain may be more useful.

If we want to understand what is driving population health we also need a variety of health indicators, perhaps especially indicators that are less sensitive to contextual change. In the example above, the ability to kneel or a test of range of motion would be less dependent on contextual change compared to ability to walk a given distance.

In conclusion, studies of health trends require a variety of health indicators that reflect multiple dimensions of health. Indicators should have varying degrees of objectivity and dependence on contextual change. The greatest challenge in trend studies is often to maintain consistency in successive waves in regards to health indicators and fieldwork in order to maintain comparability over time.

